# Histological changes of kidney in diabetic nephropathy

**Published:** 2015

**Authors:** Mohsen Pourghasem, Hamid Shafi, Zahra Babazadeh

**Affiliations:** 1 Cellular and Molecular Biology Research Center (CMBRC), Babol University of Medical Sciences, Babol, Iran.; 2 Fatemeh Zahra Infertility and Reproductive Health Research Center, Babol University of Medical Sciences, Babol, Iran.

**Keywords:** Diabetes mellitus, Nephropathy, Histological changes

## Abstract

Diabetes mellitus is the most common cause of chronic renal disorders and end-stage kidney disease in developed countries. It is the major cause of dialysis and transplantation. Failure in renal function causes wide disorders in the body. Diabetes results in wide range of alterations in the renal tissue. It is believed that early histological changes in diabetic nephropathy are detectable 2 years after diabetes is diagnosed. The glomerular alterations are the most important lesions in the diabetic nephropathy (DN). The Renal Pathology Society provides a new pathological classification for the detection of histopathology of DN. It divides diabetic nephropathy into four hierarchical glomerular lesions. Alloxan or streptozotocin induced diabetic rat is the one most widely used specie to study DN. Histological changes in the rat DN closely resemble the human disease and the most information of this review was obtained through the study of rat DN. All cell types of the kidney such as mesangial cells, podocytes and tubulointerstitial cells are liable to be affected in the event of DN. Severity of renal lesions is associated to the clinical aspect of renal outcome, but the aim of this article was only to review the histological changes of kidney in diabetes mellitus.

Diabetes mellitus is a metabolic disorder due to pancreatic dysfunction in insulin secretion and response (1). According to the International Diabetes Federation (IDF), its prevalence projected to rise from 285 million people in 2010 to 439 million in 2030, an approximate increase of 50%. In 2009, it was reported that DN is the cause of 44% of all cases of end stage renal disease (ESRD) in the United States (2). Both types of diabetes mellitus contribute greatly to health care cost and mortality due to the high incidence of nephropathy leading to ESRD, and the fact that they are a major cause of dialysis and kidney transplantation (1, 3). Several factors related to DN include the effect of genetic susceptibility, high glucose, polyol pathway activation, renin–angiotensin system activation, reactive oxygen species (ROS), activation of the protein kinase C pathway, increase of advanced glycation end-product (AGE) and glomerular hyperfiltration (4-6). It is believed that early histological changes in diabetic nephropathy are detectable 2 years after diabetes is diagnosed (7). Although with respect to histological changes, there is substantial overlap in nephropathy of type 1 and type 2 diabetes mellitus but in this paper, type 1 diabetes mellitus has been considered. Numerous methods are necessary for an accurate diagnosis of diabetes mellitus which include hematoxylin and eosin, masson trichrom, periodic acid- shiff (PAS) and periodic acid methenamine silver stains for light microscopy.

Furthermore, immunohistochemistry, electron microscope and morphometric method are also necessary. The Renal Pathology Society (RPS) provides a new pathological classification on the histopathological detection of DN (8). It divides diabetic nephropathy into four hierarchical glomerular lesions. Although the evaluation of interstitial and vascular changes has been separated, in this classification, the damage inflicted by glomerular lesions is the lowest in group one but increases throughout the groups. Glomerular alterations as most important lesions were classified as follows: class I: glomerular basement membrane thickening; class IIa: mild mesangial expansion; class IIb: severe mesangial expansion; class III: nodular sclerosis and class IV: global glomerulosclerosis in >50% of glomeruli.

Alloxan or streptozotocin-induced diabetic rat is the most widely in used studying diabetic nephropathy. Histological changes in the rat diabetic nephropathy closely resemble the human disease (9). Most of the information in this review was obtained through the study of rat diabetic nephropathy. Severity of renal lesions is associated with the clinical aspect of renal outcome but the aim of this article was only to review the histological changes of kidney in diabetes mellitus. All cell types of the kidney such as mesangial cells, podocytes and tubulointerstitial cells are liable to be affected in the event of diabetic nephropathy.

**Expression of l** **ipofuscin pigments** **: **Lipofuscin pigment storage in the renal tubular cells of rat DN that was previously reported by this author is a sign of cell injury (10). Lipofuscin pigments significantly increased in proximal convoluted tubules in the early stage of diabetic nephropathy (figure 1). It has not been yet reported for human DN. It seems that high tubular lysosomic load may induce lipofuscin storage in diabetic nephropathy (11). It is related to some parameters, for instance 1) plasma lipoproteins glycation convert their qualities and cannot be digested by tubular lysosomic enzymes, so are stored as residual bodies similar to storage disease. Glycation is a non-enzymatic reversible reaction in hyperglycemia (among sugars) and free amino groups in proteins, but over time it becomes fixed. It has been announced that early glycosylation products (EGPs) induce glomerular hyperfiltration even in normal rats. The glycation of materials influences a wide range of chemical, cellular and tissue effects leading to nephropathy (12).

2) The decrease of basement membrane glycoproteins and its negative charge leads to more protein leakage and this high load of proteins cannot be broken down into amino acids. 3) The reduction of renal barrier potency and more leakage occurs due to protein glycation in the basement membrane. Thickening of the basement membrane may occur for compensation. 4) Increasing the kidney's blood flow due to hemodynamic principle, increases the glomerular filtration and greater protein leakage to Bowman's space. 

**Figure 1:**
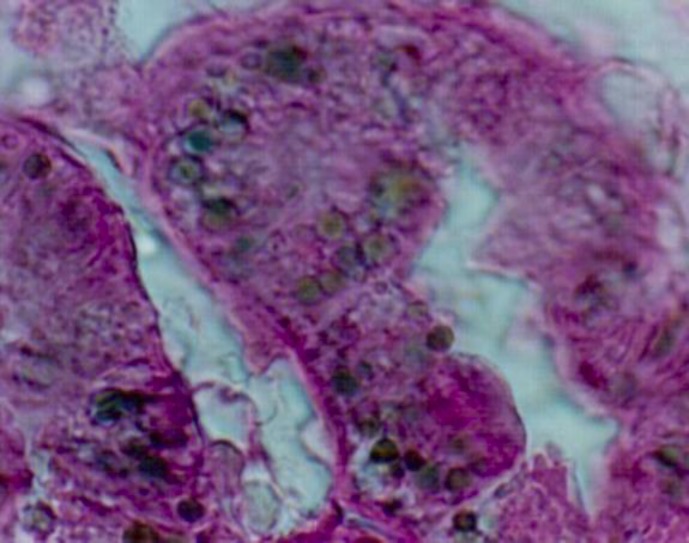
Expression of lipofuscin pigments in the proximal tubules of diabetic kidney

Schmorl’s method × 400 (10). Furthermore, it was shown that due to a lack of Vitamin E, and high fatty oxidation, lipofuscin pigments were detectable in diabetic erythrocytes (13). In vitro studies showed anti-oxidant agents in diabetic rats decreased the amount of lipofuscin pigment storage (14).

**Cellular hypertrophy and proliferation** **: **Another aspect of histological alteration in DN is cellular hypertrophy. Biological effects of the TGF-b system due to hyperglycemia in kidney cells include cellular hypertrophy and stimulation of extracellular matrix production (6). In 2000, Ziyadeh et al. reported that TGF-b1expression and bioactivity increase in tubular epithelial cells, glomerular mesangial cells, and interstitial fibroblasts in hyperglycemic culture (6). In agreement with this, Wolf et al. reported that neutralizing anti-TGF-b antibodies switch off the stimulation of collagen biosynthesis in hyperglycemia (15). Furthermore, diabetes mellitus induces proliferation in the kidney specifically in proximal tubules, which is an early hyperplasia of hypertrophy (16). 

Numerous growth factors such as insulin-like growth factor 1 (IGF-1), platelet-derived growth factor (PDGF), vascular endothelial growth factor (VEGF), and epidermal growth factor (EGF) contribute to the early proliferation of tubular system in diabetes (3). For instance Feliers in 2010 proposed that hyperglycemia and endogenous renin-angiotensin system (RAS) stimulate VEGF synthesis (17)

It is interesting to note that intrauterine hyperglycemia is accompanied by nephron default and that maternal diabetes is an important risk factor for inborn nephrogenesis. Dezfoolian in 2009 showed lower total glomerular number and total mesangial volume in diabetic offspring due to lower cortical volume (18). Mesangial matrix alteration was significantly demonstrated. Previous studies on short term diabetic nephropathy exhibited glomerular mesangial and cortical hypertrophy while long term studies displayed glomerular basement membrane thickness (18). 

**Vacuolarization of renal cells** **: **According to cell shape and function, cell vacuolization of tubules may correspond to cell adaptation in new stressful situations (hyperglycemia) and later cell damage (figure 2). It is associated to glycogen deposition or subnuclear lipid vacuolization.

**Figure 2:**
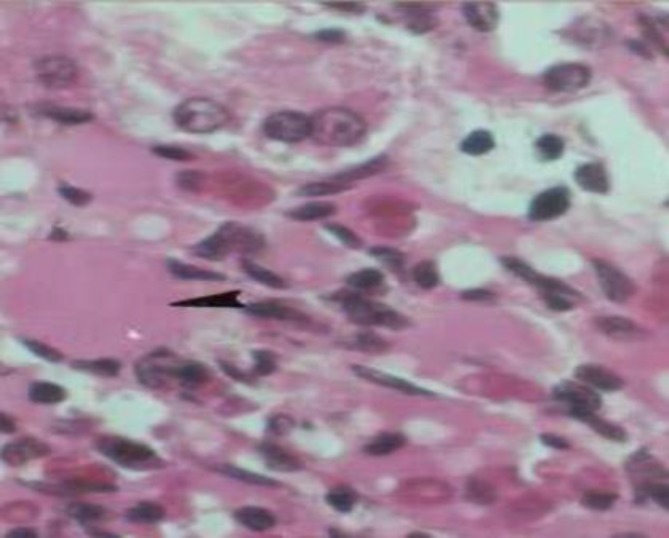
Eosinophilic deposits in the diabetic kidney (Arrow) and vacuolarization of renal cells H&E×400 (40).

Glycogen deposition in cells of tubules is referred to as Armanni-Ebstein cells which occur in severe hyperglycemia. It was demonstrated that anti-fibrotic and anti-inflammatory agents can reduce the vacuolar changes (19). 

**GAGs changes in diabetes** **: **Glycosaminoglycans (GAGs) such as heparin sulfate and chondroitin sulfate cover the luminal surface of the glomerular endothelial cells (20). These GAGs induce negative charge and are believed to be a significant component of the glomerular charge barrier (21).

 Proteoglycans include glycosaminoglycan chains and protein core that are secreted in extracellular matrix or maintained at the cell surface. GAGs are hydrated with negative charged molecules and have important roles in the barrier, meaning its deduction leads to glomerular hyperfiltration and proteinuria in diabetic nephropathy (22).

**Figure 3:**
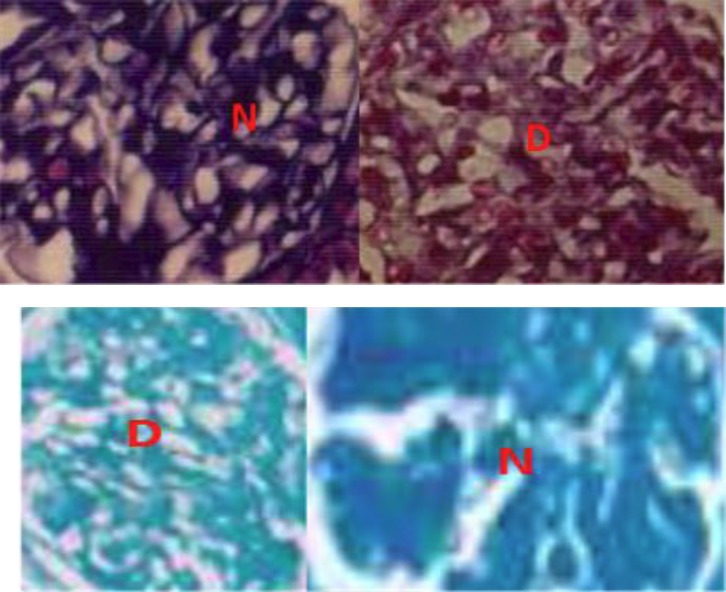
Decrease of alcian blue concentration in the diabetic glomeruli. N (nondiabetic) and D (diabetic) (23).

Hyperglycemia affects GAGs synthesis. Over 8 weeks, we demonstrated a significant decrease both in hyaluronic acid and chondroitin sulfate in the early stages of DM diabetic rats (figure 3), while there was no significant reduction in alcian blue concentration of heparan and keratan sulfate using critical electrolyte concentration CEC staining (23). However, this needs to be confirmed by the immunohistochemistry method. Vernier et al. reported heparin sulfate diminished counting anionic sites in electron micrographs (24). 

This controversy can be explained that 8 weeks was not an appropriate length of time to make heparin sulfate changes or could be detected in small quantity by CEC method. It was demonstrated that there was controversy between mesangial proliferation and GAG expression in diabetic nephropathy (25). Interestingly, GAGs alteration in diabetic mice was not restricted to the kidney, but also present in the brain tissue (26). GAGs sulfation is disrupted during hyperglycemic culture, as it has critical role in binding and activating different enzymes and proteins such as growth factors, extracellular components (collagen, laminin), chemokines and many other enzymes (lipase and protease). Therefore, the disruption of sulfated GAGs is followed by diabetes (26). Deckert formulated that defects in sulfation enzymes of HSPG (heparin sulfate proteoglycan) were considered as a possible genetic reason for global vascular dysfunction, proteinurea and diabetic nephropathy development (25). Although it has not been completely proven but there is a correlation between molecular basis of diabetic nephropathy and cardiovascular complications (27).

Locatelli stated that new GAGs antibodies such as HS (heparin sulfate) are secreted during diabetic nephropathy. These changes have also been performed in vitro under diabetic conditions (28). Furthermore several researchers demonstrated the sound prognosis of taking GAG-like production in experimental diabetic animals, which may balance synthesis and degradation of extracellular matrix (29). As a result of the same characteristic feature of diabetic nephropathy in rat and human, the therapeutic effect of GAG-like component may be realized in human diabetic nephropathy.

**Diabetic Nephropathy and Podocytes** **: **The structure of the glomerular filtration barrier includes fenestrated endothelium, glomerular basement meinbrone (GBM), podocyte foot processes and slit diaphragms. Reduction or changes of one or more elements of this complex is insufficient to the barrier and leads to proteinuria (29). Podocytes have an important and core role in the glomerular filtration barrier. Its foot processes interdigitate each other and combine with the foot processes of neighboring podocytes make a physical barrier. In DN apoptosis, loss of podocytes has been observed (30). It could be mediated by increased Smad7 (31), AGE (32), angiotensin II (33) and reactive oxygen species (34). Moreover, hyperglycemia causes detachment of podocyes from GBM. Interestingly, this type of cell can be traced in the urine of diabetic patients and it worsens as the disease progresses from normoalbuminuria to microalbuminuria and finally to macroalbuminuria (35). Hyperglycemia induces generation of reactive oxygen species (ROS) through the NADPH oxidase and ROS production initiates apoptosis of podocytes, meaning podocyte apoptosis can be reduced using NADPH oxidase inhibitor (34). Under diabetic conditions, all cell types of the kidney including endothelial cells, tubulointerstitial cells, podocytes and mesangial cells can be affected. On the other hand, any injury and dysfunction of one cell type extends to all renal cell types and affects renal function (36). 

**Increase of glomerular volume** **: **The renal structural alteration is characterized by the early glomerular and tubular hypertrophy. Glomerular lesion is the most significant alteration in DN (37). In humans, glomerural sclerosis appears in 2 forms, diffuse and nodular mesangial expansion, however, according to our studies in rats, it only appears to be diffuse and there is no report showing nodular form in other animals (38, 39). The thickening of basement membrane in glomerulus and tubules, and the progressive accumulation of extracellular matrix components are undertaken due to an increase in gene expression and protein synthesis such as collagen IV, laminin, and fibronectin (6). It was reported that these alterations lead to an increase in kidney weight (39). 

Morphometric studies showed that mesangial matrix and basement membrane thickness have close correlation to nephropathy (40). The main reason of this damage is the nonenzymatic glycosylation of plasma and glomerulus basement membrane proteins (12, 41). In hyperglycemic conditions glucose interacts covalence with amino groups of proteins in the early stage of diabetes in a way that is revisable, but by the passage of the time they will be fixed to collagen molecules. Mesangium hypertrophy includes mesangial cells hyperplasia and over secretion of ECM components. The expansion of mesangium and glomerular hypertrophy can be detected as early as 5–7 years after the onset of diabetes (42-44).

 Mesangial expansion causes the collapse of some, and later on all of the lumen of the capillaries. As a result, glomerular volume increases (40). The increase of IgG and IgM, complement C and fibrin leakage in glomerulus not only results in the presence of their sediments in ECM but also stimulates basement membrane proliferation (45). In a previous study, we discussed that ECM thickness could be a result of the secretion of collagen IV, fibronectin and laminin in hyperglycemia situation (40).

In 1994, Walkiskava stated that podocytes, mesangial and endothelial cells both secrete collagen IV in ECM, and this can be the early critical sign of diabetes mellitus (46). Furthermore, Osterby demonstrated that the increase of mesangial volume in the glomerulus of rats and human is similar (47). 

Basement membrane glycosilation causes the filtration barrier to become inefficient, results in plasma leakage, and in the proliferation of mesangial cells. Several studies described protein glycosylation as the main reason of DN complications. It was announced that 2 weeks hyperglycemic culture induced cell hypertrophy and hyperplasia because protein kinase C (PKC) activates ECM proliferation by mesangial cells (48).

 Ziyadeh in 2000 observed progressive proteinuria, and a decrease in glomerular filtration due to pathobiology of secretion of collagen IV and fibronectin in hyperglycemic situation after 10-20 weeks (6).Other alterations that are not popular include capsular drop, an abnormality in glomerular tubular junction and atubular glomeruli in DN. 

**Histological alterations in Tubulointerstitium** **: **Tubulointerstitium is composed of the tubular system, interstitial cells and vascular system, and makes up approximately 90% of kidney volume (49). Tubular hypertrophy, thickening of the tubular basement membrane and interstitial inflammation with mononuclear cell infiltration can be early histological alterations in the diabetic kidney (50, 37, figure 4). Progress of tubulointerstitium abnormalities leads to tubulointerstitial fibrosis and tubular atrophy (IFTA).

**Figure 4:**
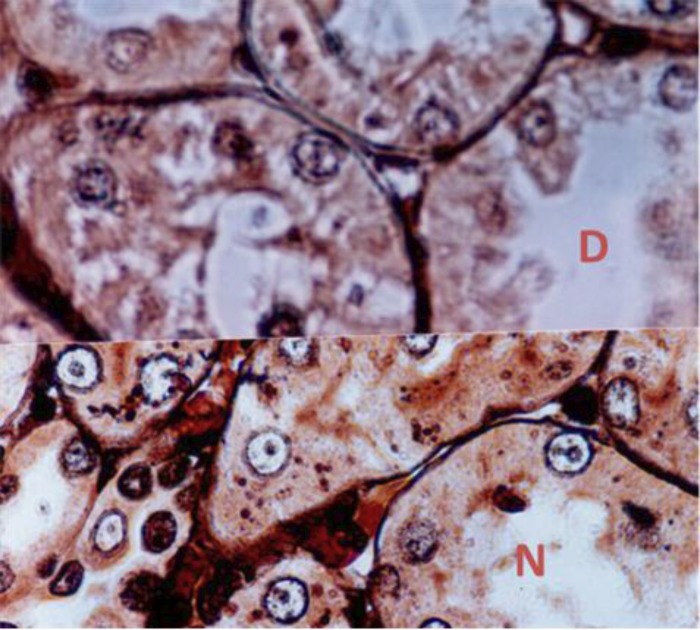
Thickening of the tubular basement membrane in the diabetic kidney. D (diabetic) and N (nondiabetic) silver nitrate × 400 .

Eosinophilic deposition is an early sign of the renal fibrosis due to glycation of extra cellular matrix (ECM) and the loosening of the balance of ECM protein synthesis and degradation (figure 2). It was reported that ECM glycation was started 6 months other diabetic initiation in diabetic nephropathy (51).

**Microcardiovascular changes in diabetic nephropathy:** Renal arteries of any size can be affected by diabetes mellitus, ( 39, 21). In diabetic nephropathy, hyalinosis occurs in both afferent and efferent arterioles but the involvement of efferent arterioles is more specific (52) (figure 4). Nitrite oxide plays a critical role in modulation of endothelial function. It is cleaned up by glucose in hyperglycemia.

**Figure 5:**
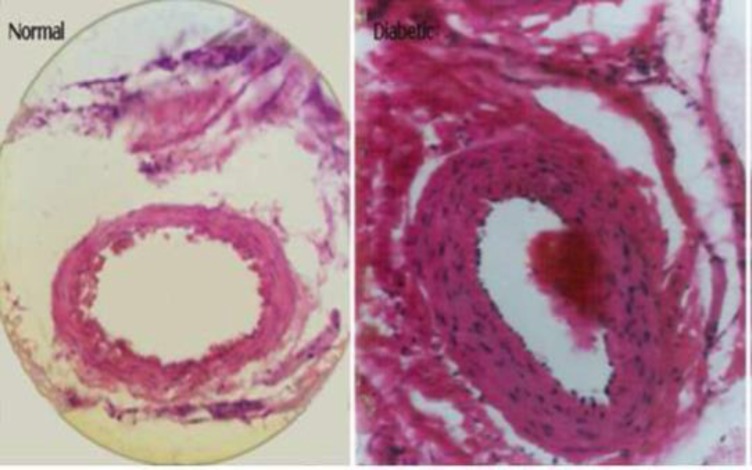
Thickening of the renal arterial wall in the diabetic kidney. H&E × 200 ( 40).

Deen et al. demonstrated this due to the important role of endothelial cells in vascular permeability, its dysfunction leads to renal and vascular pathology in diabetic nephropathy (21). 

**Figure 6:**
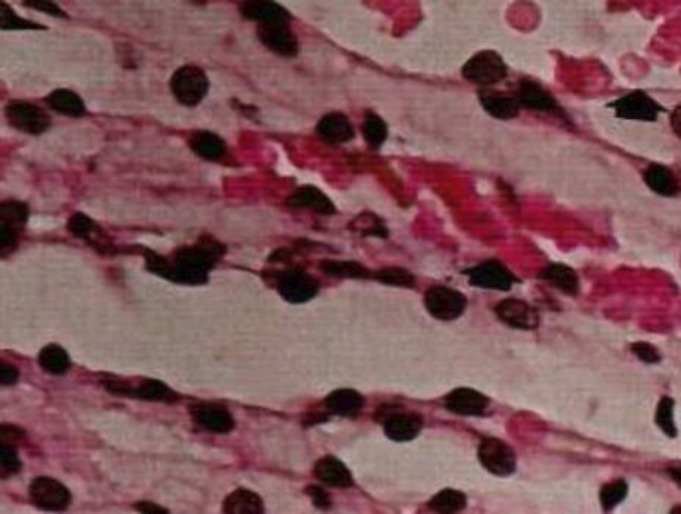
Increasing of blood flow in diabetic kidney. H&E × 400 ( 40).

Although many of these findings support this hypothesis but some data have also questioned the specificity correlation in hyperglycemia, glomerular changes and cardiovascular disorders. It has been shown that hyperfilteration is associated to vasodilation, vascular hypertrophy, damage of glomerular and tubular arteries (39) (figure 6). It was assumed that the modulation of blood pressure and hyperglycemic control are the major tools to prevent microvascular damage and further complications in diabetic kidneys (27). The renal arterial thickness is a progressive complication in diabetic kidneys which persuade hypertension and ischemia nephropathy.

Increased angiotensin 2 secretion in diabetes mellitues is the most essential evidence of arterial hypertrophy, atherosclerosis, and proliferation of smooth and mesangial cells, so angiotensin-converting-enzyme inhibitor can restrict diabetes progression (39, 53).

## References

[1] American Diabetes Association (2009). Diagnosis and classification of diabetes mellitus. Diabetes Care.

[2] Shaw JE, Sicree RA, Zimmet PZ (2010). Global estimates of the prevalence of diabetes for 2010 and 2030. Diabetes Res Clin Prac.

[3] Vallon V, Thomson SC (2012). Renal function in diabetic disease models: the tubular system in the pathophysiology of the diabetic kidney. Annu Rev Physiol.

[4] Ziyadeh FN (2004). Mediators of diabetic renal disease: the case for TGF-β as the major mediator. J Am Soc Nephrol.

[5] Yung S, Chau MK, Zhang Q, Zhang CZ, Chan TM (2013). Sulodexide decreases albuminuria and regulates matrix protein accumulation in C57BL/6 mice with streptozotocin-induced type I diabetic nephropathy. PloS One.

[6] Ziyadeh FN, Hoffman BB, Han DC (2000). Long-term prevention of renal insufficiency, excess matrix gene expression, and glomerular mesangial matrix expansion by treatment with monoclonal antitransforming growth factor-β antibody in db/db diabetic mice. Proc Natl Acad Sci U S A.

[7] Mac-Moune LF, Szeto CC, Choi PC (2004). Isolate diffuse thickening of glomerular capillary basement membrane: a renal lesion in prediabetes?. Mod Pathol.

[8] Tervaert TW, Mooyaart AL, Amann K (2010). Pathologic classification of diabetic nephropathy. J Am Soc Nephrol.

[9] Yamamoto T, Nakamura T, Noble NA, Ruoslahti E, Border WA (1993). Expression of transforming growth factor beta is elevated in human and experimental diabetic nephropathy. Proc Natl Acad Sci U S A.

[10] Pourghasem M, Jalali M, Nikravesh M, Rasoli B, Aminian N (2000). Detection of Alloxan induced Lipofuscin pigmentation in the cells of renal tubule of diabetic rats. J Babol Univ Med Sci.

[11] Forbes JM, Cooper ME, Oldfield MD, Thomas MC (2003). Role of advanced glycation end products in diabetic nephropathy. J Am Soc Nephrol.

[12] Sabbatini M, Sansone G, Uccello F (1992). Early glycosylation products induce glomerular hyperfiltration in normal rats. Kidney Int.

[13] Jain SK, Levine SN, Duett J, Hollier B (1991). Reduced vitamin E and increased lipofuscin products in erythrocytes of diabetic rats. Diabetes.

[14] Aoki Y, Yanagisawa Y, Yazaki K (1992). Protective effect of vitamin E supplementation on increased thermal stability of collagen in diabetic rats. Diabetologia.

[15] Wolf G, Hamann A, Han DC (1999). Leptin stimulates proliferation and TGF-&bgr; expression in renal glomerular endothelial cells: Potential role in glomerulosclerosis. Kidney Int.

[16] Vallon V (2011). The proximal tubule in the pathophysiology of the diabetic kidney. Am J Physiol Regul Integ Comp Physiol.

[17] Chiarelli F, Gaspari S, Marcovecchio ML (2009). Role of growth factors in diabetic kidney disease. Horm Metab Res.

[18] Dezfoolian A, Panahi M, Feizi F (2009). Stereological evaluation of renal glomeruli in offspring of diabetic female rats. Yakhteh Med J.

[19] Lau X, Zhang Y, Kelly D, Stapleton D (2013). Attenuation of Armanni–Ebstein lesions in a rat model of diabetes by a new anti-fibrotic, anti-inflammatory agent, FT011. Diabetologia.

[20] Haraldsson B, Nystrom J, Deen WM (2008). Properties of the glomerular barrier and mechanisms of proteinuria. Physiol Rev.

[21] Deen WM, Lazzara MJ, Myers BD (2001). Structural determinants of glomerular permeability. Am J Physiol Renal Physiol.

[22] Esko JD, Lindahl U (2001). Molecular diversity of heparan sulfate. J Clin Invest.

[23] Pourghasem M, Nasiri E, Sum S, Shafi H (2013). The assessment of early glycosaminoglycan concentration changes in the kidney of diabetic rats by critical electrolyte concentration staining. Int J Mol Cell Med.

[24] Vernier RL, Steffes MW, Sisson-Ross S, Mauer SM (1992). Heparan sulfate proteoglycan in the glomerular basement membrane in type 1 diabetes mellitus. Kidney Int.

[25] Deckert T, Feldt-Rasmussen B, Borch-Johnsen K, Jensen T, Kofoed-Enevoldsen A (1989). Albuminuria reflects widespread vascular damage. Diabetologia.

[26] Pourghasem M, Jorsaraee SGA, Farsi M, Soltanpour N, Kamali NA (2010). Changes of Glycosaminoglycans in the brain tissue of diabetic rats. J Gilan Univ Med Sci.

[27] Raats CI, Van Den Born J, Berden JH (2000). Glomerular heparan sulfate alterations: mechanisms and relevance for proteinuria. Kidney Int.

[28] Locatelli F, Canaud B, Eckardt K U (2003). The importance of diabetic nephropathy in current nephrological practice. Nephrol Dial Transplant.

[29] Declèves AE, Sharma K (2010). New pharmacological treatments for improving renal outcomes in diabetes. Nat Rev Nephrol.

[30] Kalluri R (2006). Proteinuria with and without renal glomerular podocyte effacement. J Am Soc Nephrol.

[31] Schiffer M, Bitzer M, Roberts IS (2001). Apoptosis in podocytes induced by TGF-beta and Smad7. J Clin Invest.

[32] Chuang PY, Yu Q, Fang W, Uribarri J, He JC (2007). Advanced glycation endproducts induce podocyte apoptosis by activation of the FOXO4 transcription factor. Kidney Int.

[33] Jia J, Ding G, Zhu J (2008). Angiotensin II infusion induces nephrin expression changes and podocyte apoptosis. Am J Nephrol.

[34] Susztak K, Raff AC, Schiffer M, Bottinger EP (2006). Glucose-induced reactive oxygen species cause apoptosis of podocytes and pdocyte depletion at the onset of diabetic nephropathy. Diabetes.

[35] Nakamura T, Ushiyama C, Suzuki S (2000). Urinary excretion of podocytes in patients with diabetic nephropathy. Nephrol Dial Transplant.

[36] Maezawa Y, Takemoto M, Yokote K (2015). Cell biology of diabetic nephropathy: roles of endothelial cells, tubulointerstitial cells and podocytes. J Diabetes Investig.

[37] An Y, Xu F, Le W (2015). Renal histologic changes and the outcome in patients with diabetic nephropathy. Nephrol Dial Transplant.

[38] Alsaad KO, Herzenberg AM (2007). Distinguishing diabetic nephropathy from other causes of glomerulosclerosis: an update. J Clin Pathol.

[39] Pourghasem M, Aminian N, Behnam Rasoli M, Nikravesh M, Jalali M (2001). Study of renal glomerular mesangium and volume changes in alloxan induced diabetic rat. Cell J (Yakhteh).

[40] Pourghasem M, Nasiri E, Shafi H (2014). Early Renal Histological Changes in Alloxan-Induced Diabetic Rats. Int J Mol Cell Med.

[41] Crowley ST, Brownlee M, Edelstein D (1991). Effects of nonenzymatic glycosylation of mesangial matrix on proliferation of mesangial cells. Diabetes.

[42] Osterby R, Hartmann A, Nyengaard JR, Bangstad HJ (2002). Development of renal structural lesions in type-1 diabetic patients with microalbuminuria Observations by light microscopy in 8-year follow-up biopsies.. Virchows Arch.

[43] Fioretto P, Steffes MW, Mauer SM (1994). Glomerular structure in non-proteinuric insulin-dependent diabetic patients with various levels of albuminuria. Diabetes.

[44] Mauer SM, Steffes MW, Ellis EN (1984). Structural functional relationships in diabetic nephropathy. J Clin Invest.

[45] Strassheim D, Renner B, Panzer S (2013). IgM contributes to glomerular injury in FSGS. J Am Soc Nephrol.

[46] Wakisaka M, Spiro MJ, Spiro RG (1994). Synthesis of type VI collagen by cultured glomerular cells and comparison of its regulation by glucose and other factors with that of type IV collagen. Diabetes.

[47] Osterby R (1992). Glomerular structural changes in type 1 (insulin-dependent) diabetes mellitus: causes, consequences, and prevention. Diabetologia.

[48] Studer RK, Craven PA, DeRubertis FR (1993). Role for protein kinase C in the mediation of increased fibronectin accumulation by mesangial cells grown in high-glucose medium. Diabetes.

[49] Gilbert RE, Cooper ME (1999). The tubulointerstitium in progressive diabetic kidney disease: more than an aftermath of glomerular injury?. Kidney Int.

[50] Najafian B, Alpers CE, Fogo AB (2011). Pathology of human diabetic nephropathy. Contrib Nephrol.

[51] Sugimoto H, Grahovac G, Zeisberg M, Kalluri R (2007). Renal fibrosis and glomerulosclerosis in a new mouse model of diabetic nephropathy and its regression by bone morphogenic protein-7 and advanced glycation end product inhibitors. Diabetes.

[52] Stout LC, Kumar S, Whorton EB (1994). Insudative lesions- their pathogenesis and association with glomerular obsolescence in diabetes: A dynamic hypothesis based on single views of advancing human diabetic nephropathy. Hum Pathol.

[53] Yoshiji H, Kuriyama S, Kawata M (2001). The angiotensin-I-converting enzyme inhibitor perindopril suppresses tumor growth and angiogenesis: possible role of the vascular endothelial growth factor. Clin Cancer Res.

